# Supplementary material for: Obstetric risk profiles and causes of death: Estimating their association with cesarean sections among maternal deaths in Mexico
Source: PLoS One. 2024 May 9;19(5):e0302369. doi: 10.1371/journal.pone.0302369 (PMC11081334; doi:10.1371/journal.pone.0302369)
Supplement: S1 Table — (DOCX) [file pone.0302369.s001.docx]

**Supplementary Table A1**. Ten most frequent immediate causes of maternal death amongst women who did not undergo a C-section in Mexico, 2010-2014.

| **Rank** | **Hypertensive disorders** | **Obstetric haemorrhage** | **Pregnancy related infection** | **Contributory conditions** | **Other obstetric complications** | **Unanticipated complications of managment** | **Non-obstetric complications (indirect)** | **Other codes of interest** |
| --- | --- | --- | --- | --- | --- | --- | --- | --- |
| 1 | Other specified general symptoms and signs (R688) (13.44%) | Shock, not elsewhere classified (R571) (43.23%) | Septicemia, unspecified (A419) (29.73%) | Septicemia, unspecified (A419) (18.68%) | Pulmonary embolism without mention of acute cor pulmonale (I269) (16.15%) | Anaphylactic shock, unspecified (T782) (30.00%) | Septicemia, unspecified (A419) (12.52%) | Septicemia, unspecified (A419) (19.53%) |
| 2 | Intracerebral hemorrhage (I619) (12.65%) | Other specified general symptoms and signs (R688) (8.59%) | Other specified general symptoms and signs (R688) (17.57%) | Shock, not elsewhere classified (R571) (16.48%) | Shock, not elsewhere classified (R571) (11.18%) | Brain death (G936) (10.00%) | Other specified general symptoms and signs (R688) (8.55%) | Failure of sterile precautions during procedure (E872) (10.06%) |
| 3 | Shock, not elsewhere classified (R571) (8.30%) | Disseminated intravascular coagulation (D65X) (6.51%) | Puerperal sepsis (O85X) (8.11%) | Other specified general symptoms and signs (R688) (10.99%) | Other specified general symptoms and signs (R688) (8.70%) | Cardiac arrest, cause unspecified (I469) (10.00%) | Acute respiratory failure (J960) (6.87%) | Shock, not elsewhere classified (R571) (6.51%) |
| 4 | Coma, unspecified (R402) (3.95%) | Cardiac arrest, cause unspecified (I469) (3.65%) | Septic shock (R572) (6.76%) | Disseminated intravascular coagulation (D65X) (5.49%) | Septicemia, unspecified (A419) (6.21%) | Coma, unspecified (R402) (10.00%) | Respiratory failure, unspecified (J969) (3.97%) | Other specified general symptoms and signs (R688) (5.92%) |
| 5 | Pulmonary embolism without mention of acute cor pulmonale (I269) (3.56%) | Failure of sterile precautions during procedure (E872) (2.86%) | Shock, not elsewhere classified (R571) (5.41%) | Pulmonary embolism without mention of acute cor pulmonale (I269) (4.40%) | Failure of sterile precautions during procedure (E872) (4.35%) | Hypovolemic shock (R570) (10.00%) | Shock, not elsewhere classified (R571) (3.97%) | Acute respiratory failure (J960) (4.14%) |
| 6 | Septicemia, unspecified (A419) (3.16%) | Septicemia, unspecified (A419) (2.34%) | Pulmonary embolism without mention of acute cor pulmonale (I269) (4.05%) | Septic shock (R572) (4.40%) | Acute respiratory failure (J960) (4.35%) | Shock, unspecified (R579) (10.00%) | Failure of sterile precautions during procedure (E872) (3.66%) | Pulmonary edema (J81X) (3.55%) |
| 7 | Subarachnoid hemorrhage, unspecified (I609) (3.16%) | Postpartum hemorrhage (O721) (2.34%) | Disseminated intravascular coagulation (D65X) (2.70%) | Failure of sterile precautions during procedure (E872) (3.30%) | Hypovolemic shock (R570) (4.35%) | Other specified general symptoms and signs (R688) (10.00%) | Hypovolemic shock (R570) (3.36%) | Intracerebral hemorrhage (I619) (2.96%) |
| 8 | Acute respiratory failure (J960) (3.16%) | Hypovolemic shock (R570) (2.34%) | Cardiac arrest, cause unspecified (I469) (2.70%) | Acute myocardial infarction (I219) (3.30%) | Obstetric embolism (O881) (3.73%) | Ill-defined and unknown cause of mortality (R99X) (10.00%) | Brain death (G936) (3.05%) | Other shock, not elsewhere classified (R578) (2.96%) |
| 9 | Brain death (G936) (2.77%) | Shock during or following labor and delivery (O751) (2.08%) | Other shock, not elsewhere classified (R578) (2.70%) | Hypovolemic shock (R570) (3.30%) | Septic shock (R572) (3.11%) |  | Intracerebral hemorrhage (I619) (3.05%) | Surgical operation and other surgical procedures as the cause of abnormal reaction of patient or of later complication, without mention of misadventure at the time of operation (E878) (2.37%) |
| 10 | Cardiac arrest, cause unspecified (I469) (2.77%) | Labor and delivery complicated by intrapartum hemorrhage, unspecified (O679) (1.82%) | Bacterial infection, unspecified (A499) (1.35%) | Cardiac arrest, cause unspecified (I469) (2.20%) | Disseminated intravascular coagulation (D65X) (2.48%) |  | Acute myocardial infarction (I219) (2.60%) | Respiratory failure, unspecified (J969) (2.37%) |
