# Supplementary material for: Obstetric risk profiles and causes of death: Estimating their association with cesarean sections among maternal deaths in Mexico
Source: PLoS One. 2024 May 9;19(5):e0302369. doi: 10.1371/journal.pone.0302369 (PMC11081334; doi:10.1371/journal.pone.0302369)
Supplement: S2 Table — (DOCX) [file pone.0302369.s002.docx]

**Supplementary Table A2**. Ten most frequent immediate causes of maternal death amongst women who had a C-section in Mexico, 2010-2014.

| **Rank** | **Hypertensive disorders** | **Obstetric haemorrhage** | **Pregnancy related infection** | **Contributory conditions** | **Other obstetric complications** | **Unanticipated complications of managment** | **Non-obstetric complications (indirect)** | **Other codes of interest** |
| --- | --- | --- | --- | --- | --- | --- | --- | --- |
| 1 | Intracerebral hemorrhage (I619) (11.83%) | Shock, not elsewhere classified (R571) (41.60%) | Septicemia, unspecified (A419) (44.07%) | Other specified general symptoms and signs (R688) (14.89%) | Pulmonary embolism without mention of acute cor pulmonale (I269) (18.56%) | Septicemia, unspecified (A419) (8.33%) | Septicemia, unspecified (A419) (9.93%) | Septicemia, unspecified (A419) (19.05%) |
| 2 | Other specified general symptoms and signs (R688) (10.25%) | Other specified general symptoms and signs (R688) (8.54%) | Other specified general symptoms and signs (R688) (16.95%) | Shock, not elsewhere classified (R571) (10.64%) | Other specified general symptoms and signs (R688) (8.38%) | Anoxic brain damage, not elsewhere classified (G931) (8.33%) | Other specified general symptoms and signs (R688) (8.80%) | Failure of sterile precautions during procedure (E872) (8.33%) |
| 3 | Coma, unspecified (R402) (6.93%) | Disseminated intravascular coagulation (D65X) (7.16%) | Shock, not elsewhere classified (R571) (5.08%) | Pulmonary embolism without mention of acute cor pulmonale (I269) (8.51%) | Acute respiratory failure (J960) (7.78%) | Cardiac arrest, cause unspecified (I469) (8.33%) | Acute respiratory failure (J960) (6.32%) | Other specified general symptoms and signs (R688) (6.55%) |
| 4 | Shock, not elsewhere classified (R571) (6.64%) | Postpartum hemorraghe (O721) (3.31%) | Disseminated intravascular coagulation (D65X) (3.39%) | Septicemia, unspecified (A419) (6.38%) | Shock, not elsewhere classified (R571) (7.78%) | Pulmonary edema (J81X) (8.33%) | Hypovolemic shock (R570) (6.09%) | Pulmonary embolism without mention of acute cor pulmonale (I269) (5.95%) |
| 5 | Subarachnoid hemorrhage, unspecified (I609) (4.04%) | Pulmonary embolism without mention of acute cor pulmonale (I269) (3.03%) | Pulmonary embolism without mention of acute cor pulmonale (I269) (3.39%) | Cardiac arrest, cause unspecified (I469) (6.38%) | Septicemia, unspecified (A419) (5.39%) | Epilepsy, unspecified (G409) (4.17%) | Shock, not elsewhere classified (R571) (5.64%) | Hypovolemic shock (R570) (4.76%) |
| 6 | Failure of sterile precautions during procedure (E872) (3.75%) | Failure of sterile precautions during procedure (E872) (2.75%) | Cardiac arrest, cause unspecified (I469) (3.39%) | Ill-defined and unknown cause of mortality (R99X) (6.38%) | Cardiac arrest, cause unspecified (I469) (4.19%) | Brain death (G936) (4.17%) | Respiratory failure, unspecified (J969) (3.16%) | Pneumonia, unspecified organism (J189) (4.17%) |
| 7 | Septicemia, unspecified (A419) (3.03%) | Labor and delivery complicated by intrapartum hemorrhage, unspecified. (O679) (2.75%) | Coma, unspecified (R402) (3.39%) | Postpartum hemorraghe (O721) (4.26%) | Obstetric embolism (O881) (4.19%) | Other postprocedural complications and disorders of nervous system, not elsewhere classified (G978) (4.17%) | Acute myocardial infarction, unspecified (I219) (2.93%) | Unknown Condition (R572) (4.17%) |
| 8 | Pulmonary edema (J81X) (3.03%) | Septicemia, unspecified (A419) (2.48%) | Septic shock (R572) (3.39%) | Hypovolemic shock (R570) (4.26%) | Hypovolemic shock (R570) (3.59%) | Intracerebral hemorrhage (I619) (4.17%) | Pulmonary edema (J81X) (2.93%) | Acute respiratory failure (J960) (3.57%) |
| 9 | Brain death (G936) (2.89%) | Acute respiratory failure (J960) (2.48%) | Acute myocardial infarction, unspecified (I219) (1.69%) | Disseminated intravascular coagulation (D65X) (2.13%) | Acute myocardial infarction, unspecified (I219) (2.99%) | Acute respiratory failure (J960) (4.17%) | Pulmonary embolism without mention of acute cor pulmonale (I269) (2.71%) | Pulmonary edema (J81X) (2.98%) |
| 10 | Acute respiratory failure (J960) (2.89%) | Other forms of shock (R578) (2.48%) | Cardiac arrest (I490) (1.69%) | Failure of sterile precautions during procedure (E872) (2.13%) | Pulmonary edema (J81X) (2.40%) | Other complications of anesthesia during the puerperium (O748) (4.17%) | Heart failure, unspecified (I509) (2.71%) | Coma, unspecified (R402) (2.98%) |
