# Supplementary material for: Obstetric risk profiles and causes of death: Estimating their association with cesarean sections among maternal deaths in Mexico
Source: PLoS One. 2024 May 9;19(5):e0302369. doi: 10.1371/journal.pone.0302369 (PMC11081334; doi:10.1371/journal.pone.0302369)
Supplement: Attachment — Submitted filename: CS and Maternal deaths PLOSONE.docx [file pone.0302369.s003.docx]

Dear Fritz, Jimena and colleagues

Thank you very much to you and your team for submitting your research for consideration for publication to PLOSONE. The paper addresses and important topic of global interest. The rising rate of caesarean section and the less-than-optimal decline in maternal mortality is a major concern globally . Indeed, existing data suggests that the world might not achieve the Sustainable Development Goal (SDG) of reducing maternal mortality to less than 70 000 per 100 000 livebirths by the year 2030 if the situation continue as is. Studies like yours is important in that they provide additional insight into some of the factors that should be looked into in the global pursuit to achieve the 2030 SDG 3.1. You provide a compelling background and justification for the study . Thank you for doing that. The paper is a bit difficult to follow as is at present and perhaps you should look at the following points to try and improve the readability of your work and also the chances of the paper being published:

1. **Title**: On reading the paper one gets the impression that the aim of the paper was to assess the association between mode of delivery, obstetrics risk factors and maternal deaths. If that is the case, you might want to revise the title to reflect this. Please refer to the PLOSONE author resource (*https://plos.org/resource/how-to-write-a-great-title/*) for guidance on how to write a winning title.

2. **Aim/Objectives**

Consider using scientific language to communicate the aim of the study and avoid using flowery words like unravel and elucidating. The reader should be very clear from reading the aim of the study as to what the study is all about. This is a bit does not clear at present. May be the aim should be to assesses the association between the mode of delivery , maternal characteristics and maternal deaths or assess the contribution of mode of delivery and maternal characteristic on maternal deaths , for example.

3. **Method**

3.1. Thanks for describing the sources of your data.

3.2. Consider writing this section in a past tense as recommended by the reviewers.

3.3. Outcome variables: It is very important to choose the outcome variables carefully to ensure that they answer the research question/ objectives. The socio-demographic and pregnancy variables chosen are appropriate. It would have been more useful to also look at co-morbid conditions as these could potentially influence maternal deaths. In other words, is it simply having a caesarean section or having a caesarean section in the background of other co-morbid conditions (or other factors) that increases the risk of maternal death? What about the experience of the surgeon and the level of care of the public healthcare facilities since majority of the deaths took place in public sector facilities. The following article might be of assistance ( *Gebhardt G S, Fawcus S, Moodley J, Farina Z. Maternal death and caesarean section in South Africa: Results from the 2011 - 2013 Saving Mothers Report of the National Committee for Confidential Enquiries into Maternal Deaths. SAMJ, S. Afr. med. j. [Internet]. 2015 Apr [cited 2023 Dec 29] ; 105( 4 ): 287-291. Available from: http://www.scielo.org.za/scielo.php?script=sci_arttext&pid=S0256-95742015000300025&lng=en.* [*http://dx.doi.org/10.7196/SAMJ.9351*](http://dx.doi.org/10.7196/SAMJ.9351)*.*)

4**. Results**

4.1. From the data, it seems like there were more deaths in the normal vaginal delivery group compared to caesarean section, but you seem to lean more on caesarean section related maternal deaths which are less compared to the normal delivery group. There is also notable significant difference between the women who died after caesarean section and those following normal delivery, but this data does not seem to be addressed by the paper. It might be worth spending some time looking and scrutinising this data as this might be something you should look include in your multimodal regression analysis.

4.2. Thank you for using the Robson classification to show the most indication for caesarean section and the risk of dying according to the Robson group.

4.3. Consider separating diagnosis and cause of death. Haemorrhage is the triggering event but what did the women die of ? Is it hypovolaemic shock? Those with pre-eclampsia did they die from bleeding , brain haemorrhage , embolism, etc? You tried to do this in Table 4 but can be further improved. Perhaps you need to scrutinise the data even further.

5. **Conclusion**

It would be appropriate to comment on this after the revised version because of the lack of alignment between the aim of the study, challenges with the interpretation and the conclusion arrived at.

6. **Additional comments**

Please see incorporate the specific comments from the reviewers in addition to the above. English language editing strongly recommended.

I hope that the peer review does not discourage you. The aim is to improve the paper and make it suitable for publication.

Looking forward to the revised version.

Kind regards

Lawrence Chauke

Academic Editor
