# Supplementary material for: Obstetric risk profiles and causes of death: Estimating their association with cesarean sections among maternal deaths in Mexico
Source: PLoS One. 2024 May 9;19(5):e0302369. doi: 10.1371/journal.pone.0302369 (PMC11081334; doi:10.1371/journal.pone.0302369)
Supplement: Attachment — Submitted filename: Response to Reviewers.docx [file pone.0302369.s004.docx]

Cuernavaca, Mexico, February 16^th^ 2023

Dr. Lawrence Chauke

Academic Editor

PLOS One

Dear Dr Chauke,

We are pleased to submit the revised version of our paper originally titled “Cesarean sections among maternal deaths in Mexico: Obstetric Risk Profiles and Other Correlates*”* (manuscript ID PONE-D-23-37685) for your consideration. As explained below, the title has changed to “Obstetric Risk Profiles and Causes of Death: Estimating their Association with Cesarean Sections Among Maternal Deaths in Mexico”.

We sincerely appreciate your input and the reviewers’ constructive criticism. We believe this has greatly enhanced and improved the presentation of our work.

Below, we respond point-by-point to specific journal requirements, your own comments and those of the reviewers. Comments appear in Italics.

Best regards,

Dr. Jimena Fritz

Corresponding Author

**Journal Requirements:**

*1. When submitting your revision, we need you to address these additional requirements.*

*Please ensure that your manuscript meets PLOS ONE's style requirements, including those for file naming. The PLOS ONE style templates can be found at* 
[*https://journals.plos.org/plosone/s/file?id=wjVg/PLOSOne_formatting_sample_main_body.pdf*](https://journals.plos.org/plosone/s/file?id=wjVg/PLOSOne_formatting_sample_main_body.pdf) *and* 
[*https://journals.plos.org/plosone/s/file?id=ba62/PLOSOne_formatting_sample_title_authors_affiliations.pdf*](https://journals.plos.org/plosone/s/file?id=ba62/PLOSOne_formatting_sample_title_authors_affiliations.pdf)*.*

We have ensured that the re-submission complies with PLOS ONE style requirements.

*2. Please note that PLOS ONE has specific guidelines on code sharing for submissions in which author-generated code underpins the findings in the manuscript. In these cases, all author-generated code must be made available without restrictions upon publication of the work. Please review our guidelines at* [*https://journals.plos.org/plosone/s/materials-and-software-sharing#loc-sharing-code*](https://journals.plos.org/plosone/s/materials-and-software-sharing#loc-sharing-code) *and ensure that your code is shared in a way that follows best practice and facilitates reproducibility and reuse.*

We will share the code upon acceptance of the article. In the meantime we have uploaded it to FigShare (<https://figshare.com>) with restrictions.

*3. Note from Emily Chenette, Editor in Chief of PLOS ONE, and Iain Hrynaszkiewicz, Director of Open Research Solutions at PLOS: Did you know that depositing data in a repository is associated with up to a 25% citation advantage (*[*https://doi.org/10.1371/journal.pone.0230416*](https://doi.org/10.1371/journal.pone.0230416)*)? If you’ve not already done so, consider depositing your raw data in a repository to ensure your work is read, appreciated and cited by the largest possible audience. You’ll also earn an Accessible Data icon on your published paper if you deposit your data in any participating repository (*[*https://plos.org/open-science/open-data/#accessible-data*](https://plos.org/open-science/open-data/#accessible-data)).

Thank you for the suggestion. We have uploaded the minimal dataset and the code to FigShare, with restrictions that will be lifted upon acceptance of the article.

*4. In the online submission form, you indicated that your data will be submitted to a repository upon acceptance.  We strongly recommend all authors deposit their data before acceptance, as the process can be lengthy and hold up publication timelines. Please note that, though access restrictions are acceptable now, your entire minimal  dataset will need to be made freely accessible if your manuscript is accepted for publication. This policy applies to all data except where public deposition would breach compliance with the protocol approved by your research ethics board. If you are unable to adhere to our open data policy, please kindly revise your statement to explain your reasoning and we will seek the editor's input on an exemption.*

Thank you for the suggestion. We have uploaded the minimal dataset and the code to FigShare, with restrictions that will be lifted upon acceptance of the article.

*5. Please include your full ethics statement in the ‘Methods’ section of your manuscript file. In your statement, please include the full name of the IRB or ethics committee who approved or waived your study, as well as whether or not you obtained informed written or verbal consent. If consent was waived for your study, please include this information in your statement as well.*

The full ethics statement has been included in the revised version, at the beginning of the Methods Section, as requested.

*6. Please ensure that you refer to Figure 1 in your text as, if accepted, production will need this reference to link the reader to the figure.*

We apologize for this omission and have included a call to Figure 1 in the revised version.

**Academic Editor Comments to Author:**

**Additional Editor Comments:**

*Dear Fritz, Jimena and colleagues*

*Thank you very much to you and your team for submitting your research for consideration for publication to PLOSONE. The paper addresses and important topic of global interest. The rising rate of caesarean section and the less than optimal decline in maternal mortality is a major concern globally . Indeed existing data suggests that the world might not achieve the Sustainable Development Goal (SDG) of reducing maternal mortality to less than 70 000 per 100 000 livebirths by the year 2030 if the situation continue as is. Studies like yours is important in that they provide additional insight into some of the factors that should be looked into in the global pursuit to achieve the 2030 SDG 3.1. You provide a compelling background and justification for the study . Thank you for doing that. The paper is a bit difficult to follow as is at present and perhaps you should look at the following points to try and improve the readability of your work and also the chances of the paper being published:*

Thank you for your kind comments, we tried our best to produce an improved version of the manuscript, incorporating your valuable suggestions. As the additional analyses requested by you and the reviewers were extensive, we decided to invite an additional co-author with both clinical and research experience, who focused both on information retrieval and interpretation of results concerning immediate causes of death, co-morbid conditions, and indications for C-sections. Please note that in our original submission, we incorrectly stated that we analyzed deaths from 2010 to 2015. Actual deaths analyzed are from the 2010 to 2014 period, with the confusion arising from the year when data was first accessed (2015). We apologize for this mistake, which has been corrected in the revised version.

*1. Title*

*On reading the paper one gets the impression that the aim of the paper was to assess the association between mode of delivery, obstetrics risk factors and maternal deaths. If that is the case, you might want to revise the title to reflect this. Please refer to the PLOSONE author resource (https://plos.org/resource/how-to-write-a-great-title/) for guidance on how to write a winning title.*

We appreciate your observation and suggest this alternative title: “Obstetric Risk Profiles and Causes of Death: Estimating their Association with Cesarean Sections Among Maternal Deaths in Mexico*”.*

*2.Research aim/objectives*

*Consider using scientific language to communicate the aim of the study and avoid using flowery words like unravel and elucidating. The reader should be very clear from reading the aim of the study as to what the study is all about. This is a bit not clear at present. May be the aim should be to assesses the association between the mode of delivery , maternal characteristics and maternal deaths or assess the contribution of mode of delivery and maternal characteristic on maternal deaths , for example.*

We have tried to clarify the objective of the work in the revised version, while trying to maintain a more sober writing style over the abstract and main text.

*3. Method*

*3.1. Thanks for describing the sources of your data*

Thank you for your kind comments.

*3.2. Consider writing this section in a past tense as recommended by the reviewers*

We have ensured that the revised version of the methods is written in past tense.

*3.3. Outcome variables: It is very important to chose the outcome variables carefully to ensure that they answer the research question/ objectives. The socio-demographic and pregnancy variables chosen are appropriate. It would have been more useful to also look at co-morbid conditions as these could potentially influence maternal deaths. In other words, is it simply having a caesarean section or having a caesarean section in the background of other co-morbid conditions (or other factors) that increases the risk of maternal death?.*

Thank you for your kind suggestion. We have re-examined the data and ascertained the presence of co-morbid conditions, specifically NCDs, as described in the updated Methods section. We found that the presence of co-morbidities (present in about 12% of women) was not significantly different between delivery modes. These results have been added to the revised paper, and are featured in Table 2. Evaluating whether these were related to risk of death, which is likely, is however outside our reach, as we lack a comparison group of women who did not die.

*What about the experience of the surgeon and the level of care of the public healthcare facilities since majority of the deaths took place in public sector facilities. The following article might be of assistance ( Gebhardt G S, Fawcus S, Moodley J, Farina Z. Maternal death and caesarean section in South Africa: Results from the 2011 - 2013 Saving Mothers Report of the National Committee for Confidential Enquiries into Maternal Deaths. SAMJ, S. Afr. med. j. [Internet]. 2015 Apr [cited 2023 Dec 29] ; 105( 4 ): 287-291. Available from:*

*http://www.scielo.org.za/scielo.php?script=sci_arttext&pid=S0256-95742015000300025&lng=en.* [*http://dx.doi.org/10.7196/SAMJ.9351*](http://dx.doi.org/10.7196/SAMJ.9351)*.)*

Thank you for your comment and the really interesting paper that you recommend us. As we know, since we have visited many health facilities in Mexico^[1],^^[2]^ , surgeons have a lot of work to do in the public sector, since the volume of patients is really high. That is one of the reasons a lot of gynecologists prefer to perform a CS section instead of waiting for hours for a vaginal and spontaneous delivery. Since they do not have a lot of health personnel nor midwifes inside the hospitals, they do not have plenty of time if an emergency occurs or if any pregnant women suddenly complicate.

*4. Results*

*4.1. From the data, it seems like the were more deaths in the normal vaginal delivery group compared to caesarean section but you seem to lean more on caesarean section related maternal deaths which are less compared to the normal delivery group.*

Yes, but the 47% contrasts with 43% of all births being by C-section in 2010^[3]^, suggesting (even though this study can not prove it) c-sections might be playing a role. In addition, newly retrieved data now featured in the paper shows that a large number of c-sections appeared to be unjustified, see response to reviewer 1’s 6^th^ comment. We have added Table 5 with data about the **indications of c-sections in these women, adding results and discussion of these findings to the revised version.**

*There is also notable significant difference between the women who died after caesarean section and those following normal delivery but this data does not seem to be addressed by the paper. It might be worth spending some time looking and scrutinising this data as this might be something you should look include in your multimodal regression analysis.*

Most of the variables which showed statistical significance in the bivariate analysis were included in the logistic regression analysis, with the exception of “Delivery Care” as it is almost perfectly correlated with the performance of C-section (i.e. all C-sections are performed by doctors). Regardless, we have expanded the discussion to comment on these significant differences, in the revised version.

*4.2. Thank you for using the Robson classification to show the most indication for caesarean section and the risk of dying according to the Robson group.*

Thank you for your kind comments.

*4.3. Consider separating diagnosis and cause of death. Haemorrhage is the triggering event but what did the women die of ? Is it hypovolaemic shock? Those with pre-eclampsia did they die from bleeding , brain haemorrhage , embolism, etc? You tried to do this in Table 4 but can be further improved. Perhaps you need to scrutinise the data even further.*

We added two supplementary tables detailing the most frequently listed immediate causes of death, by maternal cause. We now discuss about the immediate causes of death in the revised version.

*5. Conclusion: It would be appropriate to comment on this after the revised version because of the lack of alignment between the aim of the study, challenges with the interpretation and the conclusion arrived at.*

With the permission of the editor, we would like to keep our original conclusions, which we believe are now actually supported by the new findings regarding the specific indications of c-sections, as well as a more clearly expressed objective of the study-

*6. Additional comments: Please see incorporate the specific comments from the reviewers in addition to the above.*

We have done that to the best of our ability, and believe that the reviewer comments allowed us to add another layer of depth to this work.

*I hope that the review does not discourage you. Looking forward to the revised version.*

On the contrary, we are motivated by your insightful comments as well as those of the reviewers. We are happy to share the revised version and are convinced that our work has been greatly enhanced. Thank you for your consideration.

**Reviewers' Comments to Author:**

*Reviewer:1*

- *Reviewer #1: General*

*Thank you for submitting the manuscript and the intention to share clinical experience and possible solutions for the Mexican populations with the rest of readership of PLOS ONE journals.*

We thank the reviewer for his/her kind comments.

- *The title of the study “Cesarean sections among maternal deaths in Mexico: Obstetric Risk Profiles and Other Correlates” suggest that maternal death will be a major point of discussion. However, this was not the case. Perhaps consider revising the title.*

We have expanded our characterization of maternal deaths, including additional details on immediate causes of death. Additionally, we have revised the title and believe the revised version more accurately reflects the aims of our work.

- *The manuscript requires Proofreading for scientific language and grammatical errors.*

*We have tried to improve the quality of the written language in the revised version of the manuscript.*

*Introduction.*

- *Third paragraph about the trend of slowing in the decrease of maternal deaths… Please such statistics should be made available here for the reader to understand that there has been a slowdown.*

According to the Global Burden of Disease, 2019, the MMR in Mexico decreased 15% from 1990 to 2000 (71.4 to 61.7 deaths per 100,000 live births); 14 % from 2000 to 2010 (61.7 to 52.9) but only 8% from 2010 to 2019 (52.9 to 48.6). This has been added to the introduction.

- *Fourth paragraph about potential strategies…This should be noted that it cannot lead to understanding the cause as association does not equal casualty.*

The reviewer is absolutely right and we have always emphasized that no causal claims can be made based on these results. This is explicitly stated in the second sentence of said paragraph.

- *The last paragraph rationalizing the study….
  How was the deduction of the caesarean sections being unnecessary and potentially contributing to maternal deaths made?*

Although in the original version of our manuscript, we relied on Robson categories to presume some c-sections might have been unnecessary, newly retrieved data from clinical summaries presented in the revised version, show that substantial numbers of the performed c-sections were inadequately indicated. We do not intend to conclude that in any of these particular cases, the unnecessary c-section played a role in the woman’s death, but, as states in the last paragraph of the introduction, these results add to the broader discussion on the topic.

*Study design, population, and data sources*

- *This section should be written in past tense.*

We have made sure that the revised version is written in past tense.

- *There are grammatical errors that need to be corrected. I recommend proofreading by an experienced proofreader or author.*

We have made sure that grammatical errors have been corrected.

- *Sentence that begins with “Briefly the BIRMM…”, need to be reconstructed. The word "briefly" appears to be related to what the BIRMM does. Perhaps remove it as it makes the sentence lose its meaning.*

We have reworded this section of the manuscript, for clarity.

- *Use of Data and ethics: Even though such is readily accessible, permission to be used for research still need to be obtained. Therefore, this section should also indicate such. The use of retrospective data does not imply that ethics clearance is not required, kindly include this information on this section.*

We thank the reviewer for pointing this out. We have included a full ethics statement at the beginning of the revised Methods section.

- *On variables: write in past tense.*

We have made sure that this section and the rest of the Methods section is written in past tense.

- *A sentence on “years of schooling…”, this needs to be clarified. Was this about grades? It cannot be the number of years at school as that may also include those who had failed as progressing as the number of years, they are at school increases.*

The reviewer is correct, we have deleted the mention of “years” and left only “schooling”. We have clarified how this variable was categorized for the analysis using completed high school as cutoff, as this description was inaccurate in the previous version.

- *Section with “place where death occurred…”, events such as dying at home doesn’t always imply that there was no access to healthcare. Patients could have had a Pulmonary Embolus and die before transportation to hospital.*

The reviewer is correct in her/his appreciation. We have added a sentence on the limitation on using place of death as a covariate in the limitations paragraph of the Discussion (page 25 of the revised version).

*Statistical Analysis*

- *Second paragraph on logistic regression: What was the rationale for estimating the probability of a Caesarean Section being done?*

This is useful to assess whether the actual number of c-sections by risk group reflect what is expected according to the level of risk represented by the Robson categories.

*Results. There are several grammatical errors that need to be corrected.*

We have got the article proofread to identify grammatical and typographical errors.

- *Sentence “it was found that among the total number of births by c-section, 31.4% of women had a previous caesarean section, whereas in vaginal deliveries, only 17.4% had this characteristic (p<0.001)”. Does this imply "only 17.4% who delivered vaginally had previous caesarean section"?*

Yes, that is correct. We have reworded for clarity, see below.

*Table 2: Compared to the data on text, it appears that there were more maternal deaths in caesarean sections than NVDs. Please confirm and correct.*

We apologized for our previous lack of clarity, we have confirmed and corrected the data presented in the text (47% of women who died underwent a c-section) and re-written the 1s paragraph of the results section for clarity.

*Civil status of “united”, the phrase "united" may be easily understood in your country. However, the manuscript is written for distribution worldwide. Consider using a term that is easily understandable and that won’t be ambiguous in other regions.*

We have changed “civil status” to “marital status” and renamed the categories to “single” and “married or co-habiting”, following the reviewer’s advice.

*Table 2: What does Antenatal Control mean?*

We meant the timing of the “1^st^ Prenatal Care Visits”, we have switched to this term for clarity.

- *Table 2: Death site: with regards to private institutions, the current results and percentages on this table gives the impression that deaths in private sector were far less. However, this was not the case as they are being shown as percentage of whole. When you calculate private hospitals alone, the picture is difference. For example, 140 of 341 is 41%, etc. Please rephrase.*

Deaths in the private sector were indeed much less than those occurring in the public sector, but we would like to clarify this is not an estimation of risk as we are lacking denominator information for this analysis (such as live births). The figure the reviewer kindly calculated above is the percentage of women who died in private hospitals that underwent a c-section. While that is an interesting piece of information, the purpose of Table 2 was in fact to compare the distribution of covariates between groups defined by mode of delivery.

- *The sentence that reads: “… terms of the place of death, it was found that 82.4% of the deaths of women who underwent a cesarean section occurred in a public institution, while in vaginal births, it was observed in 69.1% of cases.” Kindly rephrase this sentence to restore meaning.*

This whole section has been re-written for clarity.

- *Page 15: Sentence “Based on the data, it can be inferred that private institutions (p<0.001, CI 95%: 0.54-0.65) exhibited the highest tendency to perform this surgical procedure in women who eventually died.” Please review the percentage calculated as individual institution to make a better comparison.*

We included the estimated/adjusted percentage of c-sections substituting the p-value in the figures in this paragraph, as well as re-writing for added clarity.

*Discussion*

- *Reference this section: “The stratification into Robson's groups can be beneficial in identifying the obstetric and gynecological characteristics that have the greatest impact on the practice of cesarean sections. This classification system helps in distinguishing cases where the procedure was appropriate indicated from cases where it may have been unnecessary.”*

We have cited: Robson MS. Classification of caesarean sections. Fetal and Maternal Medicine Review, 2001; 12(1): 23-39.

- *Paragraph 4: In group 4 (multiparous women without a previous uterine scar, carrying a single pregnancy in cephalic position, at or beyond 37 WOG), a high proportion of cesarean sections (67.21%) was observed…” Is there no association with hypertensive diseases in this group as there was increased C/.S rate in hypertensives?. (they could be nulliparous, singleton, no previous C/S but abruption or f/d from insufficiency)*

After a case-by-case analysis of the indications for c-sections in the sample, we only found one case in group 4 women, that was related to hypertensive disorders. Only 11% of these women died of hypertensive disorders, and the most frequent cause was obstetric hemorrhage. Of the women who died of obstetric hemorrhage in this group, 61% had a c-section. This information has been added to the text.

*The sentence “Therefore, the high rate this group also suggests a potential overuse of this surgical intervention, disregarding the favorable likelihood of spontaneous labor for multiparous women.” Without knowing the indications for caesarean sections in all these categories, it is unfair to use the phrase "overuse of this procedure”.*

We thank the reviewer for this very important observation. In our revised version, we have delved deeper into the data and have been able to establish, on a case-by-case basis, whether the indication for a c-section were presumably adequate or not. The complete results for this new analysis are shown in new Table 5 and we comment on these new results throughout the discussion section.

- *The sentence “However, recent studies indicate that a previous cesarean is not an absolute indication for performing a cesarean section”, similarly, finding or describing the indications would be most relevant. I suggest you describe the indications first before making this strong statement.*

We thank the reviewer for this important comment. As we expressed in our previous response, we have been able to assess and classify each case according to the criteria by Aranda-Neri, et al (in the revised reference list). For convenience we are showing here Table 1 from said paper, which contains the suggested classification of indications here (below), but we are unwilling to directly show it in the manuscript itself for copyright concerns. We are referring the reader to that paper instead.

- *The sentence “These findings further support the notion of an overuse of cesarean sections in Mexico, as these groups do not have inherent characteristics that necessitate a cesarean deliver”, what was reported as indications? This statement is unjust and unfair unless indications are known.*

We thank the reviewer for raising this important point. As previously stated, our revised version features a thorough analysis of the adequacy of the indications of c-sections, after further scrutiny of the available data.

- *The sentence “It is interesting to note the disparity between the findings of the present study and the study conducted by Tura et al, where they reported that the largest proportion of cesarean sections was performed in groups 3, 5 and 1, with 21.4%, 21.1%, and 19.3% respectively” The main disparity is that your study was for patients who died. Therefore, it should not surprise you. These groups are not the same and cannot be compared directly.*

The reviewer is absolutely correct. We have included a sentence indicating that these figures are not directly comparable to our study’s results, however pointing out to the fact that large numbers of the women who underwent a c-section in our sample did not have an adequate justification for the procedure.

- *The sentence “This discrepancy may indicate that, despite receiving adequate prenatal care, some cesarean sections are performed without sufficient medical justification during labor and delivery.” What were the recorded indications for caesarean section?*

We have added the most frequently recorded inadequate indications for c-section. Clearly inadequate indications amounted to at least 7% of the c-sections in this group.

- *The sentence “This observation raises concerns that a cesarean section without clear clinical justification may increase the probability of maternal death.” This statement appears to be incorrect under the context.*

We have removed the paragraph in which this phrase appeared as, after review, we judged it was unsubstantiated by our findings. We apologize for this.

- *The sentence “For instance, a study by Lisonkova et al. found that the risk of severe maternal morbidity was higher in planned cesarean deliveries compared…”, This reference looked at morbidity and not primarily mortality and therefore may not be compared directly. It was also predominantly on obese patients.*

In the revised version, we acknowledge this lack of comparability with said study.

- *The sentence “The absence of a control group of surviving women significantly hampers the ability to ascertain causal relationships between cesarean section rates and maternal mortality…”, Was it not possible to have this control group? These patients’ records are likely to be available as well.*

Unfortunately, the data we had at hand was only available for women who died. Although other dataset contains information on all birth in Mexico during the study period, they are lacking a clinical summary, which hinders the comparability to our data. Given sufficient funding, we could obtain a control group from a random sample of medical files from women who did not die. We expect to do this at some point in the future, provided funding is secured.

*Conclusion*

- *The conclusion about "overuse of caesarean sections" is not appropriate because the recorded indications at booking were disregarded during analysis of this data.*

With the new data on the indications of c-sections now featured in the manuscript, we would like to keep our original conclusions.

- *The stark contrast in C/S in private versus public sector warrant a comment about further exploration into the drivers of caesarean section in the private sector in your country or comment with available local data. This is probably not much different from other countries.*

We have expanded the paragraph about “Economic Incentives” (page 23 of the revised version), to specifically comment on this issue.

*Reviewer: 2*

- *Results section, page 8 first paragraph needs to be reviewed - grammar errors
  Table 2 - antenatal/prenatal control sounds weird, change wording*

We thank the reviewer for her/his suggestions. We have updated the terminology, for clarity.

- *Cesarean section and c-section interchanged a lot throughout the article, sometimes in the same paragraph - please review*

We have gone through the manuscript to ensure a consistent use of the terms.

1. Team training in obstetric and neonatal emergencies using highly realistic simulation in Mexico: impact on process indicators. BMC Pregnancy and Childbirth. 2014, 14:367
2. AMBAR: A midwifery-led training program to improve obstetric and neonatal practices among low-risk births in Mexico. Birth. 2022
3. Trends and projections of caesarean section rates: Global and regional estimates. BMJ Glob Health. 2021;6(6).
